# Psychological Disorder Identifying Method Based on Emotion Perception over Social Networks

**DOI:** 10.3390/ijerph16060953

**Published:** 2019-03-16

**Authors:** Tie Hua Zhou, Gong Liang Hu, Ling Wang

**Affiliations:** Department of Computer Science and Technology, School of Computer Science, Northeast Electric Power University, Jilin 132000, China; thzhou@neepu.edu.cn (T.H.Z.); 2201600508@neepu.edu.cn (G.L.H.)

**Keywords:** psychological disorder, emotion perception, sentiments distribution, social network, machine learning

## Abstract

The Institute for Health Metrics and Evaluation (IHME) has stated that over 1.1 billion people suffered from mental disorders globally in 2016, and the burden of mental disorders has continued to grow with impacts on social development. Despite the implementation of strategies for promotion and prevention in mental health WHO’s Comprehensive Mental Health Action Plan 2013–2020, the difficulty of diagnosis of mental disorders makes the objective “To provide comprehensive, integrated, and responsive mental health and social care services in community-based settings” hard to carry out. This paper presents a mental-disorder-aided diagnosis model (MDAD) to quantify the multipolarity sentiment affect intensity of users’ short texts in social networks in order to analyze the 11-dimensional sentiment distribution. We searched the five mental disorder topics and collected data based on Twitter hashtag. Through sentiment distribution similarity calculations and Stochastic Gradient Descent (SGD), people with a high probability of suffering from mental disorder can be detected in real time. In particular, mental health warnings can be made in time for users with an obvious emotional tendency in their tweets. In the experiments, we make a comprehensive evaluation of MDAD by five common adult mental disorders: depressive disorder, anxiety disorder, obsessive-compulsive disorder (OCD), bipolar disorder, and panic disorder. Our proposed model can effectively diagnose common mental disorders by sentiment multipolarity analysis, providing strong support for the prevention and diagnosis of mental disorders.

## 1. Introduction

The World Health Organization (WHO) indicated that one in four people in the world will be affected by mental or neurological disorders at some point in their lives. Earlier this decade, around 450 million people suffered from mental disorders [1], and such conditions were responsible for 62% of total Disability Adjusted Life Years (DALYs) from suicide [2], placing such conditions between the leading causes of illness and suicide globally. Furthermore, mental health disorders were widely under-reported due to mental disorders being the underlying cause [3]. A study commissioned by the World Economic Forum (WEF) predicts that mental disorders will become the biggest health cost by 2030, with global costs rising to $6 trillion each year [4]. The WHO Mental Health Action Plan 2013–2020 for the next 20 years calls for the strengthening of “Information Systems, Evidence, and Research” [5,6], which requires new developments and improvements in global mental health monitoring capabilities.

With the implementation of the WHO Mental Health Gap Action Programme (mhGAP) [7], leadership and governance for mental health have been effectively strengthened, and comprehensive mental health and social care services have been provided in low-income areas. However, the determinants, onset, and severity of mental disorders are complex, such as the interactions between Internet addiction (IA) and psychiatric co-morbidity causing more obvious health burdens [8,9,10]; the first onset of mental disorders usually occurs in childhood or adolescence, but sufferers are less likely than others to participate in surveys because of shame and the implicit self-concept [11], leading to onset and lifetime prevalence sometimes being under-reported [12]. The incidence of post-traumatic stress disorder (PTSD) in some adult patients with cancer decreased with time, but others who were initially diagnosed had a persistent or worsening mental disorder 4 years later [13,14]. Therefore, these conditions can rarely be attributed to a single factor and results in great difficulty in the effective diagnosis of mental disorders, making the prevention and follow-up hard to carry out.

Although determinants and onset are different, the symptoms of some mental disorders are similar in terms of their emotional expression [15,16,17,18]. The American Psychiatric Association (APA) deems that emotions play important roles in the diagnosis of psychological disorders [19], because patients suffering from psychological disorders are always directly correlated with negative emotions. Correspondingly, an individual feeling positive emotions toward life can also be seen as a signal of mental health [20] and indicate they are functioning well, both psychologically and socially [21].

Sentiment analysis, as one of the most important branches of Natural Language Processing (NLP), is widely applied to reviews and social media for a variety of applications, including marketing [22], examining public perceptions [23], individualized teaching [24], etc., supporting more flexible information-gathering and deeper opinion understanding [25]. Especially with the growing availability and popularity of opinion-rich resources, such as personal blogs and social software, this is being spread outside of computer science to the management sciences [26] and social sciences [27] due to its importance to business and society as a whole [28]. 

Correspondingly, the flourishing of social networks and smart mobile terminals promotes research into mental health, turning to web data sources [29] and techniques based on machine learning that are increasing used for predictive analytics in mental health [30].

Recent studies show that emotional expression in social networks has a strong correlation with mental disorders [15,16,31], providing important support for the computer-aided diagnosis of mental disorders. Most studies use the sentiment analysis of two categories’ sentiments (positive and negative) to build diagnosis models [16,17]. However, clinical descriptions of many mental disorders show more than just two sentiments according to the WHO International Classification of Diseases, 11th version (ICD-11), taking the five mental disorders of this paper as examples (listed in Table 1). Therefore, the coarse-grained sentiment analysis of mental disorders might lead to a great boundary ambiguity between mental disorders, limiting the diagnosis precision.

In this paper, we propose a mental disorder aided diagnosis model based on sentiment multipolarity analysis for short texts in social networks to detect people with high probabilities of suffering from five common adult mental disorders (depressive disorder, anxiety disorder, OCD, panic disorder, and bipolar disorder) in real time. The 8 sentiments of the NRC (National Research Council Canada)-Hashtag-Emotion-Lexicon-v0.2 [32,33] are refined into 11 sentiments in term of the Plutchik Wheel [34] by cluster analysis. We consider broader sentiment categories and more rigorous affect intensity quantification rules, so as to better identify mental disorders. To further improve the diagnosis precision of our proposed model, we use mental health scales [35,36,37,38,39] to analyze the sentiment distribution of such mental disorders, and train the model by Stochastic Gradient Descent (SGD) [40] with big data, using the Stanford Twitter sentiment corpus (http://help.sentiment140.com/) [41].

### Contributions

To further study the sentiment differences between different mental disorders, we analyze the 11-dimensional sentiment distribution of mental disorders and propose a sentiment multipolarity analysis algorithm to detect sentiment polarity and quantify the sentiment affect intensity of user’s short texts.

We propose a mental disorder aided diagnosis model to analyze the probabilities of suffering from five common adult mental disorders, which is more conducive to monitoring the mental health of social network users.

## 2. Related Work

The arrival of Web 2.0 is bringing tremendous changes in social and scientific development, and people gradually regard the network society as their spiritual community, where they can express their emotions and thoughts. Especially in years when online social media and social networking service are prevalent, people are more inclined to express their true feelings on social media, and mental health research is gradually being integrated with social networks.

Wang et al. [42] proposed a model to explore and characterize the structure of the community of people with eating disorders using Twitter data and then classify users into those with and without the disorder. The research sheds new light on how an eating-disorder community develops on social media. Coppersmith et al. [29] examined a broad range of mental health conditions in Twitter data by identifying self-reported statements of diagnosis, and they took the experimental results as evidence that examining mental health through the lens of language was fertile ground for advances in mental health. Nguyen et al. [43] found that a large number of people used online communities to discuss mental health issues, which offered opportunities for new understanding of these communities. Sumner et al. [44] explored the extent of this to determine anti-social personality traits based on Twitter. They compared the dark triad and big five personality traits of users with their profile attributes and use of language, and the result showed that there were statistically significant relationships between such variables. Wald et al. [45] applied data mining to identify the psychopathy metrics of people using information available from their Twitter accounts. The results achieved an AUC (area under the receiver operating characteristic curve) value of 0.736, which proved that it was possible to identify abnormal psychiatric states from Twitter data.

Currently, the wide application of natural language processing is arousing research attention on the interaction between mental health and sentiment analysis. Some studies used emotion as a feature and proposed a mental disorder diagnosis model.

Wang et al. [46] built a depression detection model based on sentiment analysis in a micro-blog social network. Firstly, they proposed a sentiment analysis method to calculate the depression inclination of each micro-blog. Then, they built a depression detection model based on the proposed method and 10 features of depressed users derived from psychological research. The result showed the precisions of the classifiers were all around 80%. Wang et al. [16] proposed a sentiment analysis method by utilizing vocabulary and man-made rules to calculate the depression inclination of each micro-blog, and a depression detection model was constructed based on the proposed method for detecting depressed users on social network services. The precision of the model was around 80%, and experimental results showed that the friends of patients with depression were more likely to be depressive. Coppersmith et al. [29] presented a novel method focusing on quantifying the mental health signals of four mental disorders, post-traumatic stress disorder (PTSD), depression, bipolar disorder, and seasonal affective disorder (SAD), by using the proportion of insomnia, exercise, positive sentiment, and negative sentiment tweets as features. Pedersen [47] proposed a rule-based and lightly supervised methods system, Duluth, to predict emotions in suicide notes. The experiment proved that manual corpus analysis and the use of measures of association to identify significant engrams performed better than a lightly supervised system in terms of suicidal tendency emotion, which was intended to mimic the human emotional changes in suicide.

However, these models or methods only analyze the positive and negative sentiments of mental disorders, and the differences of sentiment intensity and sentiment multipolarity distribution between different mental disorders have not been analyzed in depth, resulting in coarse-grained diagnostic results. Therefore, it is necessary to propose a more fine-grained mental disorder aided diagnosis model to better quantify the multipolarity sentiment affect intensity of short texts and analyze the multipolarity sentiment distribution, so as to further study the sentiment differences between different mental disorders and effectively diagnose mental disorders.

## 3. Data

In order to analyze the emotional characteristics of online users with mental disorders, we used Twitter as a data source to collect tweets based on timestamp information and hashtags. Specifically, we obtained tweets with mental health hashtags by searching mental-disorder-related hashtags, including #depressive disorder, #anxiety disorder, #obsessive-compulsive disorder (OCD), #bipolar disorder, and #panic disorder. Additionally, the hashtags of disorders were searched in various forms; for instance, #obsessive-compulsive disorder could also be searched with #OCD. In this way, topics related to mental disorders were easily found. Some users admitted they were diagnosed or already had a mental disorder (no recovery time), the model can easily identify whether some Twitter users have mental disorders based on self-statements, such as “I suffer with stress and anxiety disorder and often relapse into depression”. Moreover, to balance the users’ mental health in our dataset, we also collected 400 users who were considered to have no mental disorder and their tweets over a two-week period.

To further analyze the periodical emotional characteristics of the Twitter users, we used the timestamp information of a self-statement tweet as the data collection starting point, and searched for their tweets over a two-week period. The other users had recovery time, and we collected their tweets, which were tweeted when they had a mental disorder (also 2 weeks). Generally speaking, mental disorder diagnosis is needed to analyze the sentiments and behaviors of patients over a period of time, in order to make a diagnosis. Therefore, the two-week window can reflect the users’ periodical emotional tendencies and provide sufficient data. On the other hand, our goal is to detect the existence of mental disorders by analyzing the emotional characteristics of online users over time. Therefore, selecting an appropriate time window is essential.

After the data collection, we evaluated the credibility of the content of the tweets in a manual way, as it is hard to differentiate using computers if users are just talking about a mental disorder in a tweet instead of having a mental disorder. For example, in the tweet “Nearly one-half of those diagnosed with depression are also diagnosed with an anxiety disorder. #Anxiety #Awarenesspic”, the user is just talking about anxiety disorder rather than suffering from this mental disorder, while the tweet is also assigned with a hashtag of #Anxiety. So, in order to build the effective training set of the model, we needed manually evaluate the credibility of tweet content to make sure online users actually had one or some mental disorders.

The dataset was small, as tweets were only collected over two weeks, and other than depressive disorder, anxiety disorder, obsessive–compulsive disorder, bipolar disorder, and panic disorder hashtags, slang terms could have been missed in the dataset, meaning the robustness of the method is limited to a certain extent. The dataset was sampled based on hashtags, while users without hashtags were not sampled. Therefore, the model based on the training of this dataset will have better performance in classification of hashtag users, that is, it will affect the classification accuracy of non-hashtag users. On the other hand, we collected data in July 2018, so this population sampling was suitable for studying the network context of this time; other times or contexts would affect the accuracy of the method to some extent. Table 2 summarizes the number of users identified and their median number of tweets for each condition in 2 weeks.

As shown in Table 2, the dataset contained 396 users who were considered to have a mental disorder, with 5323 tweets in total, and 400 users who were considered to have no disorder, with 6683 tweets. In this dataset, few users were identified with bipolar disorder or depressive disorder. However, more users self-declared that they had bipolar disorder or depressive disorder. Through our proposed MDAD (Mental-Disorder-Aided Diagnosis) model, more users were accurately identified with mental disorders: 152 users had depressive disorder, 154 users had anxiety disorder, 25 users had OCD, 28 users had bipolar disorder, and 114 users had panic disorder. The reasons for this were that many users suffered from more than one mental disorder at the same time, but the hashtag only listed one of these. For example, a user who had depressive disorder often had panic disorder and anxiety disorder, while the hashtag only listed depressive disorder, like the tweet “I have had depression for a few years, I feel empty, worry about everyday events, and fear death”. In this Tweet, online user’s emotional characteristics correspond to depressive disorder, anxiety disorder, and panic disorder, while the word “depression” is distinctly detected by hashtag, making anxiety disorder and panic disorder hard to detect.

## 4. Motivation

Modern people are often under psychological pressure from work and life, which causes some people’s mental health to be threatened. The burden of mental disorders continuously increases, not only impacting daily life, human rights, and economic consequences, but also affecting physical health [48,49]. Therefore, the monitoring of mental health plays a key role for mental disorder prevention, diagnosis, and treatment.

**Figure 1:**
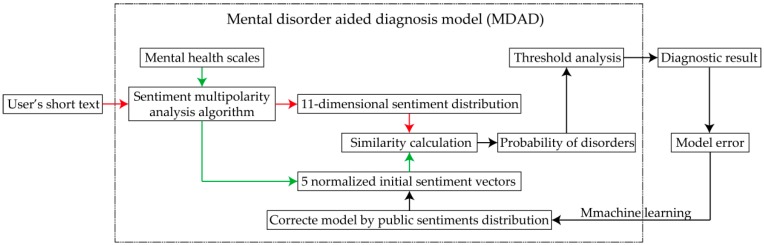
Flowchart of diagnosis model.

Our proposed MDAD can efficiently analyze the 11-dimensional sentiment distribution of five common adult mental disorders by analyzing the sentiment polarity and affect intensity of users’ short texts on social media. Firstly, we propose a sentiment multipolarity analysis algorithm to analyze sentiment polarities and quantify the affect intensities of mental health scales in order to calculate the 11-dimensional sentiment distributions of five mental disorders, and the sentiments distributions of such conditions can be treated as five normalized initial sentiment vectors. Secondly, the 11-dimensional sentiment distribution of users’ short texts can be obtained by our sentiment multipolarity analysis algorithm. Finally, the users’ probabilities of suffering from mental disorders can be obtained through sentiment distribution similarity calculation. Moreover, the MDAD is further trained by machine learning, which means that the five normalized initial sentiment vectors are corrected by public sentiment distribution according to the model error, as shown in Figure 1. On this basis, users with high probabilities of suffering from these five mental disorders can be detected in real time in order to effectively monitor their mental health, providing opportunities for timely and effective prevention, diagnosis, and follow-up.

## 5. Sentiment Multipolarity Analysis Algorithm

In this section, we propose an effective algorithm to automatically analyze sentiment polarities and quantify the affect intensities of short texts in order to calculate 11-dimensional sentiment distributions, which is important to identify users whose probability of suffering from mental disorders is high. By summarizing sentiments that people with mental disorders often express, we refine the NRC-Hashtag-Emotion-Lexicon-v0.2 into 11 sentiments according to the Plutchik Wheel by cluster analysis, and design more rigorous affect intensity quantification rules in terms of the characteristics of emotion expression and the proximity rule of qualifiers, in order to improve the accuracy of sentiment analysis. On this basis, the 11-dimensional sentiment distribution can be obtained by detecting the sentiment polarity and quantifying the sentiment affect intensity of short texts. Table 3 lists the main symbols and their definitions.

### 5.1. Multipolarity Sentiment Affect Intensity Lexicon

In this section, we rebuild the NRC-Hashtag-Emotion-Lexicon-v0.2 (NRC-Lexicon) into a multipolarity sentiment affect intensity lexicon (MSAI-Lexicon) by one-dimensional clustering and synonym analysis, so as to improve the applicability of the sentiment affect intensity lexicon.

Firstly, we apply K-means cluster algorithm to decompose the emotion lexicon, and the intensity of each emotional word is treated as a sample point. Specifically, we randomly select K cluster centers in each type of sentiment word, K = 2, and calculate the Euclidean distance between sample points as followed:

(1) $$dis(x_{i},x_{j})=Int(x_{i})-Int(x_{j}),$$

where *x_i_* denotes the *i*th sample point, *x_j_* denotes the *j*th sample point, *Int*(*x_i_*) denotes the intensity of *x_i_*, *Int*(*x_j_*) denotes the intensity of *x_j_*, and dis(*x_i_*, *x_j_*) denotes Euclidean distance between *x_i_* and *x_j_*.

According to the Euclidean distance between the sample points, the sample points are divided into class clusters that are close to the centers, and the centers are recalculated based on the mean value of cluster sample points. Repeat the calculation of Euclidean distance between sample points and the calculation of centers until algorithm convergence (centers no longer update), as follows:

(2) $$\mathrm{Cost}=\sum_{i}^{N}({\mathrm{argmin}}_{k}{\left\|x_{i}-c_{k}\right\|}^{2}),$$

where Cost denotes the loss function, *N* denotes the number of sample point, *x_i_* denotes *i*th sample point, and *c_k_* denotes *k*th center.

We select three negative sentiments in the NRC-Hashtag-Emotion-Lexicon-v0.2 (“disgust”, “sadness”, and “fear”) as a one-dimensional cluster to detect the boundaries between sub-negative-sentiments. The reason for selecting these three sentiments is that people suffering mental disorders often express more negative emotions, and the clustering result of anger and feeling balanced. Then, the 8 sentiments of the NRC-Hashtag-Emotion-Lexicon-v0.2 are divided into 11 categories, including anger, loathing, disgust, grief, sadness, surprise, terror, fear, trust, joy, and anticipation.

Secondly, WordNet [50] is selected as the dictionary for synonym matching, and the synonyms of sentiment words in the multipolarity sentiment affect intensity lexicon is re-selected and added into the multipolarity sentiment affect intensity lexicon if the sense similarity is bigger than 0.7.

Finally, each sentiment polarity word set of the multipolarity sentiment affect intensity lexicon needs to be normalized to 0–1; specific sentiment categories are shown in the Table 4.

### 5.2. Affect Intensity Quantification Rules

To obtain more accurate 11-dimensional sentiment distribution, one key issue is to establish more rigorous sentiment affect intensity quantification rules. In this section, we detail the rules, which are established according to the characteristics of emotional expression, grammar, and the proximity relationship of qualifiers between sentiment words and qualifiers.

Firstly, we assign the degree adverb weight to 0–2 depending on the modifier degree, which can enhance or weaken the affect intensity of sentiment words. For instance, with two sentences “I am very sad today” and “I am a little sad today”, the corresponding weights of “very” and “little” are 1.5 and 0.5.

Secondly, we assign the weights of emoticons and sentiment punctuation to 0–1, because emoticons and sentiment punctuation can also enhance or weaken the affect intensity of sentiment words. The difference is that emoticons and sentiment punctuation have a weaker affect intensity than degree adverbs.

Thirdly, the weight of negative adverbs is assigned to −1 because of its inversion principle to a sentence.

Finally, the sentiment transition effect of adversatives is taken into account, so that we unify a weight of 1.5 weights to the sentiment transition segment; more specifically, a weight of 1.5 will be assigned to clauses that come after the adversative. For instance, “Nice dress, but too mediocre for me” contains the adversative “but”, and although the part “Nice dress” has a joy sentiment, the clause “too mediocre for me” has a stronger disgust sentiment.

### 5.3. Sentiments Distribution

**Figure 2:**
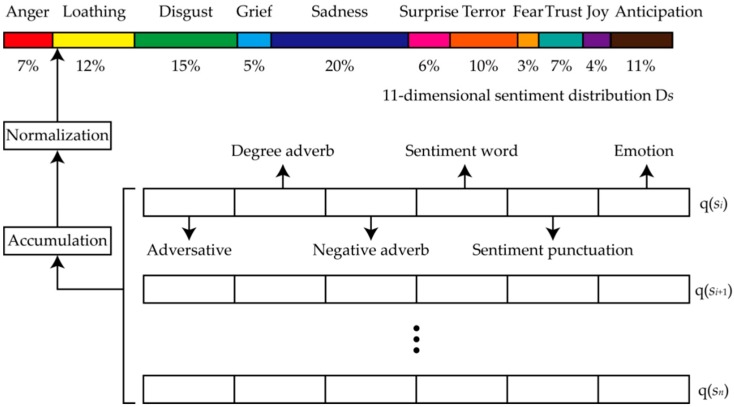
Eleven-dimensional sentiment distribution.

In this section, the 11-dimensional sentiment distribution of short texts can be obtained, as shown in Figure 2.

Step 1: Sentiment word matching. In a general context, the degree adverb, negative adverb, adversative, emoticon, and sentiment punctuation of each sentiment word are closest to it. Therefore, we match the sentiment word in the MSAI-Lexicon, and take each sentiment word as a benchmark to count and seek the closest degree adverb, negative adverb, adversative, emoticon, and sentiment punctuation.

Step 2: Sentiment affect intensity quantification. To quantify the sentiment of each sentence in a short text, we calculate its sentiment affect intensity quantification value as follows:

(3) $$q\left(s_{i}\right)=\sum_{j=1}^{n^{*}}(w_{j}\times \prod_{k=1}^{m}\alpha_{k}\times \beta \times \eta +\gamma +\tau ),$$

where *s_i_* denotes the *i*th sentence, q(*s_i_*) denotes the sentiment affect intensity quantification value for sentence *s_i_*, *w_j_* denotes the *j*th sentiment word of sentence *s_i_*, *α_k_* denotes the *k*th degree adverb, *η* denotes negative adverb, *β* denotes adversative, *γ* denotes emoticon, *τ* denotes sentiment punctuation, n* denotes the number of sentiment words, and *m* denotes the number of adverbs.

Step 3: Sentiment affect intensity accumulation. To obtain the sentiment affect intensity quantification value a of whole short text, we add up the sentiment affect intensity quantification value of each sentence as follows:

(4) $$V=\sum_{i=0}^{n}q\left(s_{i}\right),$$

where *V* denotes sentiment affect intensity vector of short texts, and q(*s_i_*) denotes the *j*th polarization sentiment quantification value.

Step 4: Eleven-dimensional sentiment distribution calculation. To avoid the effects of excessive sentiment on the diagnosis, we normalize the sentiment affect intensity vector of a short text into 11-dimensional sentiment distribution D*s*.

## 6. Mental Disorder Aided Diagnosis Model

In this section, we propose a mental disorder aided diagnosis model based on our proposed sentiment multipolarity analysis algorithm. To further improve the diagnosis precision, our proposed Mental-Disorder-Aided Diagnosis model (MDAD) is trained with tweets by Stochastic Gradient Descent (SGD), which means that the five normalized initial sentiment vectors are corrected by public sentiment distribution according to model error.

### 6.1. Normalized Initial Sentiment Vectors

In this section, we apply our proposed sentiment multipolarity analysis algorithm to analyze sentiment polarities and quantify the affect intensities of five mental health scales [34,35,36,37,38], in order to study the 11-dimensional sentiment distributions of five mental disorders.

First, the degree level of each item in a mental health scale is selected as highest, because we suppose the most severe mental disorder as a reference. Then, the contents of five mental health scales are analyzed by our algorithm separately, and the sentiments distributions of these five conditions are denoted as 5 normalized initial sentiment vectors. Finally, the normalized initial sentiment vectors of depressive disorder, anxiety disorder, obsessive–compulsive disorder (OCD), bipolar disorder, and panic disorder are defined as *Vs*(*Dd*), *Vs*(*Ad*), *Vs*(*OCD*), *Vs*(*Bd*), and *Vs*(*Pd*).

### 6.2. Similarity Calculation

The normalized initial sentiment vector *Vs*(*c*) can be treated as the emotional signal of people suffering from severe mental disorders, and the 11-dimensional sentiment distribution of a user’s short texts (D*s*) is the real emotional signal of the user, which means that users may have more than one mental disorder. To diagnose a user’s mental health, the similarity calculation of *Vs*(*c*) and D*s* is defined as the probability of suffering from the mental disorder *c*, and Prob(*c*) can be calculated as follows:

(5) $$\mathrm{Prob}(c)=\frac{\sum_{1}^{n}\left(Vs(c)[i]\times Ds[i]\right)}{\sqrt{\sum_{1}^{n}{\left(Vs(c)[i]\right)}^{2}}\times \sqrt{\sum_{1}^{n}{\left(Ds[i]\right)}^{2}}},$$

where Prob(*c*) denotes the probability of suffering from the mental disorder *c*, *Vs*(*c*)[*i*] denotes the *i*th sentiment ratio of *Vs*(*c*), and D*s*[*i*] denotes the *i*th sentiment ratio of D*s*.

### 6.3. High-Risk User Identification

In this section, we divide the probabilities of suffering from a mental disorder into three levels to separate users with a severe mental disorder, moderate mental disorder, and no or mild mental disorder. The user is treated as higher risk user if his probability of suffering from the mental disorder level is higher than 60%, and the model diagnostic result is positive.

**Definition** **1.** *(SMU): User is defined as SMU if his Prob(c) is higher than 80%.*

**Definition** **2.** *(MMU): User is defined as MMU if his Prob(c) is between 60% and 80%.*

**Definition** **3.** *(NMU): User is defined as NMU if his Prob(c) is lower than 60%.*

### 6.4. Model Training

**Figure 3:**
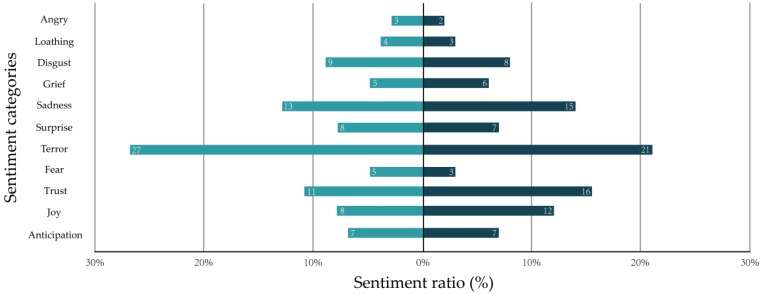
Deviation of sentiment distribution between the mental health scale and social network short texts.

However, there may be a certain deviation from the sentiment distribution of mental health scale to the sentiment distribution of users’ short texts because the language expression of the short texts on the social network is not suitable for the mental health scale analysis. For instance, in Figure 3, the sentiment distribution of the panic disorder scale is shown on the left, while the sentiment distribution of a user’s short texts is shown on the right, which means that application deviation leads to the diagnostic error of the model. Therefore, it is necessary to train the model with real social network data to further reduce diagnostic error, and so we train MDAD by machine learning, which means that the 5 normalized initial sentiment vectors are corrected by public sentiments distribution according to model error.

Public emotion can often affect a person’s emotion even in a network environment, which can be seen as the network mental neighborhood effect [51]. For instance, as most people are happy discussing a movie on Facebook, the joy sentiment of others is easy to be awakened, generally. In the same way, when many people express a sentiment on social networks, others are easily affected by these sentiments. On the other hand, when the ratio of a sentiment is high, the infectious effect of this sentiment will not be improved significantly unless its sentiment ratio increases significantly. To validate our algorithm, the datasets were assigned as shown in Table 5:

In Table 5, the datasets are divided into two parts, training data and testing data. The dataset collected is based on hashtags, and 70% of it is assigned for the training set and 30% for the testing set. Specifically, Stanford Twitter sentiment corpus training data is used to calculate average sentiments distribution, sentiment ratio, and sentiment increment (in Step 2 to Step 4), Hashtag-based training data was used to calculate diagnostic error rate, so as to optimize model parameters. Hashtag-based testing data is used to validate the diagnostic performance of MDAD.

**Figure 4:**
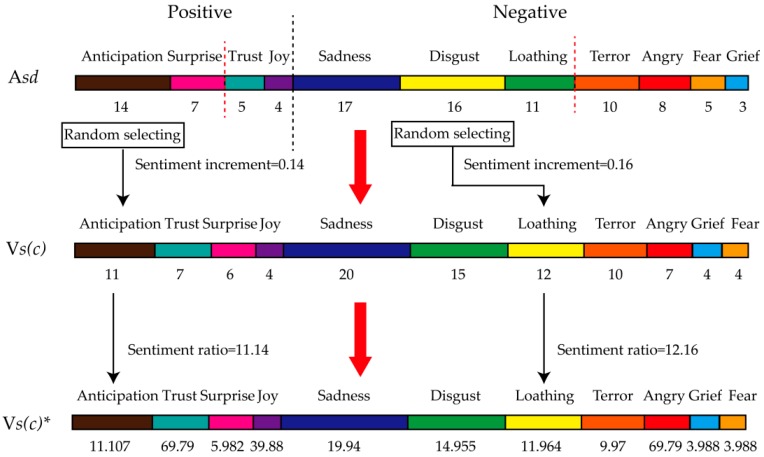
Flowchart of step 2–step 6.

Based on this, as shown in Figure 4, our model training is detailed as follows:

Step 1: Choosing one of the five mental disorders normalized initial sentiment vectors *Vs*(*c*).

Step 2: Adding 1000 users of Stanford Twitter sentiment corpus training data into the training dataset in order to calculate average sentiment distribution A*sd*.

Step 3: The 11 sentiments of A*sd* are divided into two categories (positive and negative), so as to sort sentiment into sub-sentiment distribution according to the sentiment ratio.

Step 4: One of the top two positive sentiments is randomly selected in order to calculate the sentiment increment (learning rate) by S*r*/100. In the same way, one of the top three negative sentiments is randomly selected to calculate sentiment increment.

Step 5: Adding sentiment increments to corresponding sentiment ratios of *Vs*(*c*), and the sentiment distribution of this mental disorder is normalized as *Vs*(*c*)*.

Step 6: Calculating the diagnostic error rate *Err* of *Vs*(*c*) and diagnostic error rate Err* of *Vs*(*c*)*. If *Err* > Err*, *Vs*(*c*) = *Vs*(*c*)*.

Step 7: Repeating step 2 to step 6 until the end of the iteration.

Step 8: Repeating step 1 to step 7 until the end of the iteration.

Our MDAD is trained by randomly selecting the top sentiments to calculate learning rates. The learning rates are not fixed, which resulted in the training result not necessarily being the optimal solution, but reducing the vibration time in the later stage of convergence. At this point, although the accuracy of the model is reduced, the diagnostic timeliness of MDAD is improved. In order to validate the diagnostic performance, we apply MDAD, which was trained by Stanford Twitter sentiment corpus training data and Hashtag-based training data ten times each, to measure the average diagnostic precision of five conditions with Hashtag-based testing data (119 users with condition and 120 users with no condition), as shown in Section 7.2. The average diagnostic precision of five conditions is defined as followed:

(6) $${adp}^{j}=\sum_{i=0}^{n}\frac{c_{i}(h)+c_{i}(n)}{n\times {Users}_{j}},$$

where *j* denotes the *j*th iterative, *adp^j^* denotes the *j*th average diagnostic precision of five conditions, *i* denotes the *i*th mental disorder, *n* denotes the number of mental disorders, *c_i_*(*h*) denotes number of users correctly classified as having the *i*th mental disorder, *c_i_*(*n*) denotes number of users correctly classified as not having the *i*th mental disorder and *Users_j_* denotes number of users of the *j*th iterative.

On this basis, the object function of MDAD is defined as *Max*(*adp*), and the model is iteratively optimized by error rate feedback (in Step 6). That is, the model is optimized by judging if the error rate is reduced due to the update of the sentiment distribution in this iteration. If the error rate is reduced, the update is retained and continues to iterate; if not, the update is not retained and continues to iterate, until the maximum number of iterations is reached.

## 7. Experiments

In this section, we first compared the performance of our proposed sentiment analysis algorithm with Citius [52] and SeNTU [53]. Then, we evaluated the diagnosis precision of MDAD, and performed a statistical analysis of Twitter users.

All experiments were conducted on a Windows10.0.15063 server with one CPU (i7-7700k 4.20GHz) and 1-T of main memory. Citius, SeNTU, and MDAD were implemented in Java as single-threaded programs.

### 7.1. Evaluation of Sentiment Analysis Algorithm

Our model was based on analyzing the emotional characteristics of online users’ mental disorders to diagnose whether they have mental disorders or not; the diagnostic accuracy of the model mainly depends on the performance of sentiment analysis. Therefore, we compared the sentiment analysis performance of MDAD, Citius, and SeNTU. Citius was a sentiment classifier based on naïve-bayes, with good performance in detection of two polarity categories: positive and negative. SeNTU was a Twitter sentiment analysis system developed by combining a rule-based classifier with supervised learning. It had high accuracy in classification of positive, negative, or neutral tweets.

To evaluate the performance of our sentiment analysis algorithm, we applied the measures “Precision”, “Recall”, and “F1-Measure”, which emphasized the performance of the algorithm on the analysis capability and degree of confidence. To better understand the concepts of the three measures, we introduce the concept TP (True Positive), FP (False Positive), TN (True Negative), and FN (False Negative), as listed in Table 6.

As shown in Table 6, Tp denotes the classified as true labels that are true labels, FP denotes the classified as true labels that are false labels, FN denotes the true labels that are classified as false labels, and TN denotes the false labels that are classified as false labels. The “Precision”, “Recall”, and “F1-Measure” can be defined as follows:

(7) $$\{\begin{array}{l} \mathrm{Precision}=\frac{\mathrm{Tp}}{\mathrm{TP}+\mathrm{FP}}\\ \mathrm{Recall}=\frac{\mathrm{Tp}}{\mathrm{TP}+\mathrm{FN}}\\ \text{F1-Measure}=\frac{2\times \mathrm{Precision}\times \mathrm{Recall}}{\mathrm{Precision}+\mathrm{Recall}} \end{array},$$

The higher Precision means the labels that were classified were more often true labels, and the higher Recall means more of the true labels were classified. The F1-Measure is the harmonic mean of Precision and Recall, and the higher F1-Measure means better classification performance. 

The “Precision”, “Recall”, and “F1-Measure” of MDAD, Citius, and SeNTU were calculated respectively, as listed in Table 7.

As shown in Table 4, our proposed sentiment multipolarity algorithm had higher “Precision”, “Recall”, and “F1-Measure”, proving its performance. Most sentiment words were able to be searched in our lexicon, ensuring the precise identification of sentiment. Our method overcame the ambiguity of sentiment analysis to some extent, because we applied a fine-grained sentiment lexicon. In addition, we considered the sentiment transition effect of adversatives, and carried out suitable weight distribution of synonyms for sentiment words, degree adverb, negative adverbs, adversatives, emoticons, and sentiment punctuation.

### 7.2. Model Performance Evaluation

In this section, we evaluated the performance of MDAD by the average diagnostic precision with iterative training, and the diagnosis performance was proved by the average diagnostic precision of five common mental disorders, as shown in Figure 4. Twitter data was collected based on hashtags, described in Section 3.

**Figure 5:**
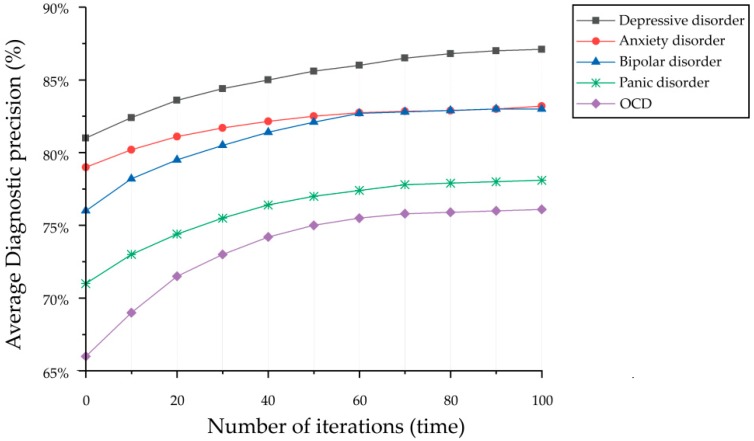
Model iterative optimization.

We applied SGD with big data to optimize the MDAD. To evaluate the performance of the MDAD, we measured the average diagnostic precision of 5 conditions ten times each, and the diagnostic performance was proved by the final average diagnostic precision. As shown in Figure 5, the average diagnostic precision increased gradually with 100 cycles of iterative optimization and eventually converged. The final average diagnostic precision of MDAD was depressive disorder at 87.3%, anxiety disorder at 83.1%, obsessive-compulsive disorder (OCD) at 76.3%, bipolar disorder at 77.8%, and panic disorder at 82.6%. The precision was the lowest for OCD and bipolar disorder, at 76.3% and 77.8%, respectively, which were impacted by the size of the sampling data. In general, the sampling of mental disorders does impact the indicators study metrics, which means training the model with a larger dataset could improve the diagnostic accuracy of the model to some extent. However, the size of the sampling data is not the main factor impacting the diagnostic accuracy of the model. Because the clinical manifestations of these two mental disorders are not only in the sentiment distribution, such as the clinical manifestations of OCD being characterized by the presence of persistent obsessions or compulsions, or most commonly both. Obsessions are repetitive and persistent thoughts, images, or impulses and urges that are intrusive, unwanted, and are commonly associated with anxiety. This compulsion is not only reflected in the sentiment distribution, but also in the behavioral characteristics of patients. Similarly, bipolar disorder is characterized by alternation with depressive disorder, leading to misdiagnosis of the disorder. Therefore, the aid analysis of behavioral characteristics is also the premise to improve the diagnostic accuracy.

Furthermore, we made a statistical analysis of our dataset on user proportions of different degrees of mental disorder (severe mental disorder, moderate mental disorder, and no or mild mental disorder).

From Table 8, the mental health of the dataset was displayed clearly. Users with severe depression and anxiety disorder had the highest percentages, which were 8.5% and 10.3%, respectively. Users with severe obsessive-compulsive disorder (OCD) had the lowest percentage of 0.9%, and bipolar disorder was the next lowest at only 2%. On the one hand, it was possible this resulted from the insufficient sample size. On the other hand, we considered that users with severe obsessive-compulsive disorder were hard to diagnose by sentiment analysis alone, and that information of behavior and repeatability were also required.

## 8. Conclusions

In this paper, we studied the relationship between mental disorders and the multipolarity sentiment distribution of social network users to propose a mental disorder aided diagnosis model (MDAD) based on sentiment multipolarity analysis. The MDAD allowed us to identify users whose probability of suffering from five common adult mental disorders was high to determine if users had a mental disorder. Additionally, we proposed a sentiment multipolarity analysis algorithm to automatically analyze sentiment polarities and quantify the affect intensities of short texts in order to calculate the 11-dimensional sentiment distributions through our rebuilt multipolarity sentiment affect intensity lexicon, which eliminated the ambiguity of sentiment analysis to a certain extent.

Our model could effectively monitor the mental health of social network users in real time, providing opportunities for timely and effective prevention, diagnosis, and follow-up. However, the average diagnostic accuracy of bipolar disorder and obsessive-compulsive disorder indicates that the precision of the MDAD was limited to further improvement due to insufficient sampling data. Therefore, we would try to train our model on a larger dataset in the future. Even so, the diagnosis result was also able to provide doctors with a reference to diagnose mental disorders of patients by reviewing their mood swings, providing an auxiliary method for smart human healthcare.

Advances in natural language processing and machine learning make the prospect of the large-scale recognition of social network short texts a near-future possibility. Among future directions, we hope to further understand the relationship between behavior and mental disorder, and incorporate user behavior into our analysis model. We are also interested in tracking the diffusion of mental disorders in populations to better understand the mental disorder neighborhood effect in social networks.

## Figures and Tables

**Table 1:** Mental disorders and clinical descriptions.

Diseases	Clinical Descriptions
Depressive disorder	Characterized by depressive mood (e.g., sad, irritable, empty) or loss of pleasure accompanied by other cognitive, behavioral, or neurovegetative symptoms that significantly affect the individual’s ability to function.
Anxiety disorder	Characterized by marked symptoms of anxiety, manifested by either general apprehension or excessive worry focused on multiple everyday events, together with additional symptoms, such as muscular tension or sleep disturbance.
OCD (Obsessive-Compulsive Disorder)	Characterized by the presence of persistent obsessions or compulsions, or most commonly both. Obsessions are repetitive and persistent thoughts, images, or impulses and urges that are intrusive, unwanted, and are commonly associated with anxiety.
Bipolar disorder	Defined by the occurrence of manic, mixed, or hypomanic episodes or symptoms. These episodes typically alternate over the course of these disorders with depressive episodes or periods of depressive symptoms.
Panic disorder	Panic attacks are discrete episodes of intense fear or apprehension accompanied by the rapid and concurrent onset of several characteristic symptoms.

**Table 2:** Description of the dataset.

Condition	Users	Median	Total
Depressive disorder related	75	16	1194
Anxiety disorder related	143	12	1711
OCD-related	32	10	316
Bipolar disorder related	16	18	289
Panic disorder related	130	14	1813
No mental disorder	400	17	6683

**Table 3:** Definition of main symbols.

Symbol	Definition
*w_j_*	*j*th sentiment word of sentence *s_i_*
*α_k_*	*k*th degree adverb
*η*	Negative adverb
*β*	Adversative
*γ*	Sentiment punctuation
*τ*	Emoticon
q(*s_i_*)	Sentiment quantification value of sentence *s_i_*
D*s*	Eleven-dimensional sentiments distribution of user’s short texts
Prob(*c*)	Probability of suffering from mental disorder *c*
*Thres*	Screening threshold of mental disorder

**Table 4:** Sentiment categories and affect intensity ranges. MSAI: multipolarity sentiment affect intensity.

8 Sentiments in NRC-Lexicon	11 Sentiments in MSAI-Lexicon
Anger	Anger
Disgust	Loathing Disgust
Sadness	Grief Sadness
Surprise	Surprise
Fear	Terror Fear
Trust	Trust
Joy	Joy
Anticipation	Anticipation

**Table 5:** Datasets assignment.

Training Data	Testing Data
Stanford Twitter sentiment corpus training data (100,000 users)	Hashtag-based testing data (119 users with condition and 120 users with no condition)
Hashtag-based training data (277 users with condition and 280 users with no condition)	

**Table 6:** Sentiment analysis performance.

	Relevant	Non-Relevant
**Retrieved**	TP (True Positive)	FP (False Positive)
**Not retrieved**	FN (False Negative)	TN (True Negative)

**Table 7:** Sentiment analysis performance.

	Precision	Recall	F1-Measure
Our method	0.77	0.92	0.84
Citius	0.69	0.79	0.74
SeNTU	0.75	0.82	0.78

**Table 8:** Mental disorder proportions of different degrees.

Condition	NMU	MMU	SMU
Depressive disorder	81.0%	10.5%	8.5%
Anxiety disorder	80.7%	9.0%	10.3%
OCD	96.9%	2.2%	0.9%
Bipolar disorder	96.5%	1.5%	2.0%
Panic disorder	85.7%	5.0%	9.3%
